# *Mannheimia haemolytica*–associated fibrinonecrotizing abomasitis in lambs

**DOI:** 10.1177/03009858241235393

**Published:** 2024-03-05

**Authors:** Estela Pérez, Francisco A. Uzal, Ricardo de Miguel, Ana Rodríguez-Largo, Raúl Reséndiz, Nicolás Streitenberger, Melissa Macías-Rioseco, Álex Gómez, Natalia Calvo-Sánchez, Marta Pérez, Lluís Luján, Javier Asín

**Affiliations:** 1 University of Zaragoza, Zaragoza, Spain; 2 AgriFood Institute of Aragon, Zaragoza, Spain; 3 University of California-Davis, San Bernardino, CA; 4 Anapath Services GmbH, Liestal, Switzerland; 5 University of California-Davis, Tulare, CA; 6 University of California-Davis, Davis, CA

**Keywords:** abomasitis, fibrinonecrotizing, immunohistochemistry, lambs, *Mannheimia* haemolytica, PCR

## Abstract

*Mannheimia haemolytica*–associated abomasitis has been clinically described as a cause of sudden death in lambs, but it is poorly characterized. We describe the pathological features of a severe fibrinonecrotizing abomasitis in 3 lambs that died suddenly. All 3 abomasums had a thickened submucosa due to edema and necrotic areas delimited by bands of degenerate neutrophils with slender nuclei (oat cells) and angiocentric distributions. The overlying mucosa was congested. Myriads of gram-negative coccobacilli were observed within the oat cell bands. *M. haemolytica* was isolated from the abomasum in all 3 animals and was serotyped as A2 in one of them. Pericarditis and pleuritis were observed in 2 of the lambs. *Clostridium* spp. were isolated in 1 lamb and detected by immunohistochemistry in the 3 animals, suggesting clostridial co-infection. *M. haemolytica* should be considered among the differential diagnoses of necrotizing abomasitis in lambs.

*Mannheimia haemolytica* is the most prevalent agent inducing acute fibrinonecrotizing bronchopneumonia and pleuritis with massive economic impact in cattle and sheep.^16^ The most important *M. haemolytica* virulence factor is the leukotoxin, a repeat in toxin that is secreted by all serotypes and induces lysis of ruminant leukocytes. Additional virulence factors include a lipopolysaccharide and the polysaccharide capsule, among others.^3^ *M. haemolytica* and *Bibersteinia trehalosi* have been associated with septicemia in lambs subjected to stressful events.^3,6,11^ Septicemia due to *M. haemolytica* tends to affect lambs younger than 2 months, while *B. trehalosi* usually affects lambs older than 4 months.^6,11^

Most authors associate *B. trehalosi* with a septicemic condition in weaned lambs known as septicemic pasteurellosis (SP).^3,5,6^ Lambs with *B. trehalosi* septicemia have short clinical courses or die with no premonitory signs. These animals display necrosis and ulceration of the upper alimentary tract, abomasum, and/or intestines, and these lesions may act as the bacterial portal of entry into the bloodstream leading to systemic dissemination and development of multifocal hepatitis, arthritis, pericarditis, leptomeningitis, and choroiditis.^3,5,6,11,12^ Some authors also associate *M. haemolytica* with SP,^11^ while others recognize a distinct *M. haemolytica–*induced septicemia (MIS) with no classic pneumonic lesions but edematous lungs and occasional pleuritis/pericarditis, serosal petechiation, lymphadenomegaly, and multifocal hepatic necrosis.^4,5,6^*M. haemolytica* is also responsible for a localized leptomeningitis in lambs.^2,6^ Reports of localized gastrointestinal lesions associated with *M. haemolytica* are scant and include cases of acute abomasitis in lambs and a calf.^7,16^ To the best of our knowledge, a form of localized abomasal mannheimiosis in lambs has not been described in any major veterinary pathology journal or textbook. We present here 3 cases of fibrinonecrotizing abomasitis associated with *M. haemolytica* in lambs.

The carcasses of 3 unrelated lambs (cases 1–3) were submitted to the necropsy service of the University of Zaragoza, Spain (case 1), and the California Animal Health and Food Safety Laboratory System, University of California-Davis, USA (cases 2 and 3). Cases were submitted in February (case 2), March (case 3), and July (case 1) of three different years. Case 1 was a 20-week-old, Rasa Aragonesa male that was kept in a research farm and died before any experimental procedure was performed. Clinical signs prior to death included a short period of depression, isolation, recumbency, drooling, labored breathing, and abdominal distension and pain. Case 2 died suddenly a few days after being moved to a green pasture, and no other signalment or clinical information was available. Case 3 was a 2-week-old, Dorper male from a 70-sheep flock that was found dead after a short episode of abdominal distension.

Necropsies were performed within 24 hours of death in all cases. Histology sampling and other ancillary tests are detailed in Supplemental Materials. Briefly, formalin-fixed tissues, including the abomasum and all major organs, were processed routinely and stained with hematoxylin and eosin; selected sections of abomasum were stained with Gram and Grocott’s methenamine silver stains. Immunohistochemistry for *M. haemolytica* OmpA^PH278^ and several *Clostridium* spp. were performed as previously described.^1,8,13^ Aerobic and anaerobic cultures were performed on samples from abomasum and other organs and complemented with molecular techniques for bacterial identification and serotyping; polymerase chain reaction (PCR) for *Clostridium septicum* was performed in fresh or formalin-fixed, paraffin-embedded abomasum. Additional tests included fecal floatation parasitologic exam and a liver mineral analysis.

At necropsy, all lambs had watery and hemorrhagic abomasal contents, severely thickened walls with transmural edema, a dark-red mucosa, and fibrinous peritonitis with subserosal echymoses and loose fibrin strands that extended to adjacent viscera (Fig. 1a). Abomasal subserosal hemorrhages and mucosal ulceration were confined to pyloric and/or fundic regions (cases 2 and 3) or were diffuse in the most severe case (case 1). Thickness of the wall was up to 2.5 cm at the edge of abomasal folds (case 1; Fig. 1a). Embedded within the abomasal wall, a focal, 1.5 cm in diameter, yellow-white abscess was found in one case (case 3). Thirteen trichophytobezoars measuring 1–2 cm in diameter were found admixed within the hemorrhagic contents of the abomasal lumen of case 1. Gross changes in other organs included focal fibrinous pericarditis and pleuritis (case 2) with extensive epicardial ecchymoses (case 1). The pulmonary parenchyma was hyperemic and slightly mottled, but otherwise unremarkable, in all three cases.

**Figure 1.**
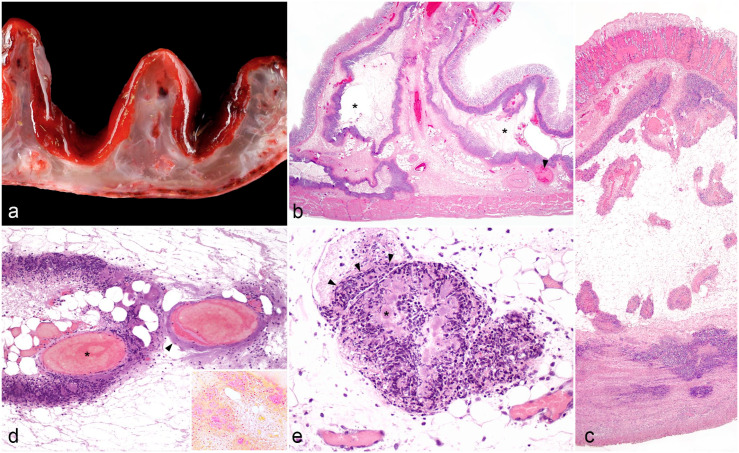
Fibrinonecrotizing abomasitis, abomasum, lamb. (a) A macroscopic view of a transverse section reveals severe thickening of the wall with submucosal edema and congested submucosal and subserosal vessels. Intact mucosa is deep red. Case 1. (b) Subgrossly, the abomasal submucosa contains multiple areas of edema, necrosis, and fibrin delimited by serpiginous bands of inflammatory cells parallel to the muscularis mucosa and internal tunica muscularis and, occasionally, to the serosa (sequestra, asterisks); gas bubbles are also embedded within these areas. The mucosa remains intact, and the submucosal vessels are congested with occasional thrombi (arrowhead). Case 2. Hematoxylin and eosin (HE). (c) The submucosa exhibits marked edema and vasculitis, and the inflammatory rim of the sequestrum adjacent to the muscle layers infiltrates muscle fibers of the internal tunica muscularis. The non-ulcerated mucosa is congested at its base with an overlying band of ischemic necrosis on the surface. Case 3. HE. (d) Two congested submucosal vessels. One vessel (asterisk) is associated with bands of karyorrhectic debris and colonies of coccobacilli that partially infiltrate the wall. Intramural colonies replace the wall of the other vessel (arrowhead). Case 1. HE. Inset: colonies of coccobacilli are gram-negative. Case 1. Gram stain. (e) Lymphatics are distended by an accumulation of degenerate neutrophils admixed with colonies of coccobacilli in the lumen (asterisk). The colonies and degenerate neutrophils infiltrate the wall (arrowheads). Adjacent venules are intact. Case 1. HE.

Histologically, all lambs exhibited fibrinonecrotizing abomasitis with very similar characteristics. The submucosa was markedly thickened by edema, fibrin, and areas of necrosis. Degenerate neutrophils with slender nuclei, morphologically compatible with oat cells, were organized in serpiginous bands parallel to the muscularis mucosa and internal tunica muscularis and surrounding central areas of necrosis (Fig. 1b, c). The oat cell bands infiltrated the internal tunica muscularis and reached the serosa in multiple areas (Fig. 1c). Occasionally, the neutrophilic infiltration within the submucosa surrounded small and large vessels (Fig. 1d). Congestion and occasional perivascular hemorrhages were common in submucosal arteries and arterioles. Most of the overlying abomasal mucosa remained intact with occasional areas of coagulative necrosis (Fig. 1b, c). Bacterial colonies occasionally infiltrated and even replaced the wall of medium to large veins and venules (Fig. 1d). Occasionally, blood vessels had fibrin thrombi (Fig. 1b), and a few lymphatic vessels contained emboli of degenerate neutrophils intermixed with bacterial colonies (Fig. 1e). The bands of oat cells were intimately associated with colonies of coccobacilli (Fig. 2a–c). Case 2 also had moderate emphysema within the submucosa (Fig. 1b). The intramural abscess in case 3 had palisades of oat cells around colonies of coccobacilli and areas of mineralization surrounded by multinucleated giant cells in the capsule.

**Figure 2.**
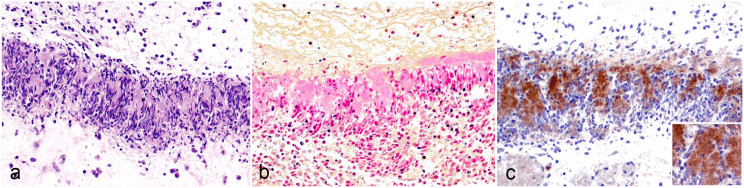
Fibrinonecrotizing abomasitis, abomasum, lamb. (a) Submucosal inflammatory band composed of a palisade of oat cells intermixed with colonies of coccobacilli. Case 1. HE. (b) Bacterial colonies are gram-negative. Case 3. Gram stain. (c) Immunohistochemistry with *Mannheimia haemolytica* OmpA^PH278^ antibody revealed immunolabelled colonies of coccobacilli within the bands of inflammation. Case 1. *M. haemolytica* OmpA^PH278^ immunohistochemistry. Inset: Higher magnification of the immunolabeled organisms.

In all cases, bands of oat cells with bacterial colonies and fibrin were attached to and infiltrated the serosa of the adjacent abdominal organs (rumen, omasum, reticulum, small and large intestines, spleen, and liver). In cases 2 and 3, there was multifocal fibrin deposition in the thoracic cavity, including on the pleura, pericardium, epicardium, and thymus. In addition, there was mild congestion and mononuclear interstitial infiltrates in the lungs of the three lambs.

The colonies of coccobacilli associated with the lesions were gram-negative in all three lambs (Figs. 1d [inset], 2b) and immunolabeled for *M. haemolytica* OmpA^PH278^ (performed in case 1, only; Fig. 2c). Gram-positive bacilli were only detected in the mucosa (cases 2 and 3). Grocott’s methenamine silver stain was negative (case 2). Immunohistochemistry to identify several *Clostridium* spp. was variably positive in all 3 animals, and *C. septicum* PCR was negative in all cases (Table 1).

**Table 1.** Summary of most relevant microbiology, molecular, and immunohistochemistry results on abomasum, lungs, and liver of 3 lambs With *Mh*-associated abomasitis.

No.	Culture				FAT—Ab	PCR—Ab			IHC—Ab					
	Organ	Aerobic	Anaerobic	YICE	Csep	MhA2	Csep	Psor	Mh	Csep	Psor	Cper	Cch	Cnov
1	Ab	Mh^a^	Neg	n/d	n/d	+	-^b^	n/d	+	+	-	+	+	+
	Lun	n/d	n/d	n/d										
	Liv	n/d	n/d	n/d										
2	Ab	Mh^a^	Cper Psor Fnec	-	+	n/d	-^c^	n/d	n/d	+	+	+	-	-
	Lun	Mh^d^ Ecoli^e^	-	n/d										
	Liv	-	-	n/d										
3	Ab^f^	Mh	n/d	n/d	n/d	n/d	-^c^	n/d	n/d	+	w+	+	-	-
	Lun	Mh	n/d	n/d										
	Liv	Mh	n/d	n/d										

Abbreviations: Ab, abomasum; Cch, *Clostridium chauvoei*; Cnov, *Clostridium novyi;* Cper, *Clostridium perfrigens*; Csep, *Clostridium septicum*; Ecoli, *Escherichia coli*; FAT, fluorescence antibody test; Fnec, *Fusobacterium necrophorum*; IHC, immunohistochemistry; Lun, lung; Liv, liver; n/d, not done; Mh, *Mannheimia haemolytica*; No, number; PCR, polymerase chain reaction; Psor, *Paeniclostridium sordellii*; w+, weakly positive; YICE, *Yersinia sp.* isolation-cold enrichment; +, positive; -, negative.

a Mixed cultures with *E. coli* and *Streptococcus* sp., both considered of no significance.

b PCR from fresh tissue.

c PCR from formalin-fixed, paraffin-embedded tissue.

d Isolated from pleural swab.

e Isolated from lung swab.

f Swab from the intramural abscess.

*M. haemolytica* was consistently isolated from the abomasal walls of the 3 lambs, including the intramural abscess of case 3 (Table 1). In case 1, the *M. haemolytica* isolate from the abomasum was serotyped as A2. *M. haemolytica* was also isolated from the pleura of case 2 and from the lung and liver of case 3. Abundant coccidian oocysts were detected in the feces of case 2, which also had a slightly reduced hepatic copper concentration (21 ppm [reference range 25–100 ppm]). All other ancillary test results were negative or within acceptable ranges.

MIS is poorly described in the scientific literature, and there is no a clear consensual association of *M. haemolytica* with SP or any gastrointestinal lesion in ruminants.^3,11^ This report demonstrates that *M. haemolytica* can elicit localized abomasitis, with secondary pericarditis and pleuritis in the absence of the classically described bronchopneumonia. The mild mononuclear infiltrates observed in the lungs of the three lambs were most likely unrelated to *M. haemolytica* infection and interpreted as incidental. Endocarditis, pleuropericarditis, and peritonitis with no pulmonary or abomasal lesions were reported in a 12-week-old Suffolk wether.^10^ Upper alimentary tract ulceration typical of SP was not seen in any of the animals of our study or in the Suffolk wether, suggesting a different pathogenesis from those previously described.^3,5,6,11,12^

The signalment and clinical history of these three animals resemble those reported for lambs with *M. haemolytica* or *B. trehalosi* septicemia, and hence, similar predisposing factors may have played a role. All lambs died suddenly or after a short clinical course, which is compatible with most cases of *Pasteurellaceae* infections.^3,6^ Lambs between 3 and 4 weeks of age are especially predisposed to MIS,^17^ which matches with the age of one of the cases in the study (case 3). However, other reports have also described outbreaks of MIS in up to 6-month-old lambs.^15^ In fact, *M. haemolytica* A2 was isolated from the 20-week-old lamb (case 1) submitted at the beginning of July. This is in agreement with the most prevalent serotype detected in a set of outbreaks of MIS, which was especially seen in May and July.^15^ Interestingly, and as commonly described in cases of SP, one of the animals died within a few days of being placed on pasture (case 2), so dietary stress could have been a trigger.^6^ Irritant trichophytobezoars may have predisposed to abomasitis in case 1, as they have been statistically associated with abomasal ulceration and hemorrhages.^18^ One of the lambs (case 3) had an intramural abscess enclosed by fibrosis, which may be indicative of a focus of restricted inflammation that subsequently became more active, perhaps due to the action of stressors.

Using bacteriological, molecular, and/or immunohistochemical analyses, *M. haemolytica* was identified in the fibrinonecrotizing abomasitis of all lambs of this study. Some of the histological features in the abomasum were reminiscent of the classic pneumonic disease caused by *M. haemolytica*, including edema, vascular damage, and formation of palisades of oat cells delimiting central areas of necrosis, similar to sequestra.^3,11^ The severity of the abomasal lesions in the absence of other remarkable findings suggests that the abomasum was the primary site of inflammation that may have led to death by toxemia and/or sepsis in these lambs. Pericarditis and/or pleuritis are usual MIS findings and are especially consistent with *M. haemolytica* A2 infections.^6^ The lymphatic system serves as a conduit between pericardial, pleural, and peritoneal serosae and may explain the observed pleuropericarditis in two of the lambs (cases 2 and 3) of this report.^19^

Braxy is an abomasitis caused by *C. septicum* that affects weaned lambs during the first year of life. This gram-positive bacillus is also an opportunistic organism that causes lesions similar to those described in our lambs, except it is associated with a much greater involvement of the mucosa, including ulceration.^14^ Emphysema could be also a distinct feature of clostridial infection, and in case 2, the emphysema was likely produced by co-infecting clostridia as *M. haemolytica* is not a gas-producing bacteria.^17^ Other clostridia, such as *Paeniclostridium sordellii*, could also be considered among the differential diagnoses of this disease process and as an opportunistic or secondary contributors in some of the lambs of this study.^9^ If these microorganisms are frequently associated with *M. haemolytica* infection, this might in part explain why *M. haemolytica* abomasitis is underdiagnosed as it might be masked by the severe necrotizing effect of the clostridial toxins.

It is worth noting that this is a retrospective work in which we pooled cases from different institutions over several years that were handled by different pathologists. As such, the diagnostic workup differed among these 3 cases, which is a common limitation of these kinds of reports. However, the consistent isolation of *M. haemolytica* coupled with very similar microscopic features is a commonality in the 3 lambs of our report.

In conclusion, *M. haemolytica* can contribute to a severe, acute fibrinonecrotizing abomasitis in lambs with secondary peritonitis and pleuropericarditis. It is unknown if some specific *M. haemolytica* virulence factors may play a role in the development of this presentation, and this should be the focus of future work if similar cases are encountered. *M. haemolytica* should be considered as a differential diagnosis in cases of fibrinonecrotizing abomasitis in lambs.

## Supplemental Material

sj-docx-1-vet-10.1177_03009858241235393 – Supplemental material for Mannheimia haemolytica–associated fibrinonecrotizing abomasitis in lambs Supplemental material, sj-docx-1-vet-10.1177_03009858241235393 for Mannheimia haemolytica–associated fibrinonecrotizing abomasitis in lambs by Estela Pérez, Francisco A. Uzal, Ricardo de Miguel, Ana Rodríguez-Largo, Raúl Reséndiz, Nicolús Streitenberger, Melissa Macías-Rioseco, Álex Gómez, Natalia Calvo-Sánchez, Marta Pérez, Lluís Luján and Javier Asín in Veterinary Pathology
