# Supplementary material for: Mannheimia haemolytica–associated fibrinonecrotizing abomasitis in lambs
Source: Vet Pathol. 2024 Mar 5;61(4):604–8. doi: 10.1177/03009858241235393 (PMC11264574; doi:10.1177/03009858241235393)
Supplement: sj-docx-1-vet-10.1177_03009858241235393 – Supplemental material for Mannheimia haemolytica–associated fibrinonecrotizing abomasitis in lambs [file sj-docx-1-vet-10.1177_03009858241235393.pdf]

Supplemental Materials

Mannheimia haemolytica-associated fibrinonecrotizing abomasitis in lambs

Estela Pérez, Francisco A. Uzal, Ricardo de Miguel, Ana Rodríguez-Largo, Raúl Reséndiz, Nicolás Streitenberger, Melissa Macías-Rioseco, Álex Gómez, Natalia Calvo-Sánchez, Marta Pérez, Lluís Luján, Javier Asín

Supplemental Materials and Methods

Abomasum and all major tissues including omasum, reticulum, rumen, esophagus, lung, pulmonary lymph nodes, trachea, diaphragm, brain, heart, small intestine, cecum, colon, spleen, liver, adrenal gland, skeletal muscle, kidney, tongue, mesenteric lymph nodes, thymus, and trachea were collected and fixed by immersion in 10% neutral-buffered formalin during 24-48 hours, embedded in paraffin, and processed routinely to produce 4 μm thick sections stained with hematoxylin and eosin. Sections of abomasum were also stained with Gram (cases 1-3) and Grocott's methenamine silver stains (case 2), and subjected to immunohistochemistry (IHC) for *Mannheimia haemolytica* detection (case 1) using a rabbit anti-outer membrane protein A (OmpA)^{PH278} antibody for sheep strains (University of Glasgow, Glasgow, UK), as described.[5,9] No positive control was used. The primary antibody was substituted by Dako REAL Antibody Diluent (Agilent, Santa Clara, CA) in the negative control. IHC against several *Clostridium* spp. (*C. septicum, C. perfringens, C. chauvoei, C. novyi, and Paeniclostridium sordelli*) using rabbit (*C. perfringens*; GenWay Biotech Inc, San Diego, CA) and goat (all the rest; VMRD, Pullman, WA) polyclonal antibodies were conducted (cases 1-3) following the California Animal Health and Food Safety laboratory system (CAHFS) standard operating procedures (SOPs) and as described.[1] Positive controls included bovine tissues from animals that had been previously inoculated with each of the clostridial species tested. The primary

antibody was substituted with non-immune serum of the corresponding species in the negative controls. Abomasum was subjected to additional microbiologic and molecular tests following CAHFS SOPs or as indicated: aerobic (cases 1-3) and anaerobic (cases 1 and 2) bacterial cultures with matrix-assisted laser desorption/ionization-time of flight mass spectrometry, *Yersinia* sp. cold enrichment culture (case 2), fluorescent antibody test for *C. septicum* (case 2), polymerase chain reaction (PCR) for *C. septicum* from fresh abomasum (case 1; performed with *q-PCR EXOone C. septicum* kit [Exopol SL, Zaragoza, Spain] following the manufacturer instructions) or from formalin-fixed, paraffin-embedded abomasum (cases 2 and 3; targeting the flagellin gene of *C. septicum* and as described[8,10,11]), PCR for *C. perfringens* from fresh abomasum (case 1; performed with q-PCR EXOone *C. perfringens* kit [Exopol SL, Zaragoza, Spain] following the manufacturer instructions), and PCR serotyping of one of the *M. haemolytica* isolates (case 1; performed with multiplex q-PCR EXOone *M. haemolytica* types A1, A2, A6 *kit* [Exopol SL, Zaragoza, Spain] following the manufacturer instructions).[2] Ancillary tests in other organs were performed following CAHFS SOPs and/or as indicated, and included aerobic/anaerobic cultures of lung (cases 2 and 3), pleura (case 2), liver (cases 2 and 3) and small intestine (case 1); PCR for *C. perfringens* in fresh intestine (case 1; performed with q-PCR EXOone *C. perfringens* kit [Exopol SL, Zaragoza, Spain] following the manufacturer instructions); *Salmonella* sp. culture from colon contents (case 3) and *Salmonella* sp. reverse transcription PCR screen and culture from feces (case 2) as described;[3,6] *rotavirus* A antigen enzyme-linked immunosorbent assay from intestinal contents (case 3) as described;[7] fecal flotation (case 2); acid-fast stain in fecal smears (case 3); and liver mineral screen (cases 2 and 3) including arsenic, cadmium, copper,

iron, lead, manganese, mercury, molybdenum, selenium, and zinc by inductively coupled plasma-mass (selenium) or atomic emission (all the rest) spectrometry as described.[4]

References

1. Abreu CC, Blanchard PC, Adaska JM, et al. Pathology of blackleg in cattle in California, 1991-2015. *J Vet Diagn Invest*. 2018;30**:** 894-901.
2. Arnal JL, Fernández A, Vela AI, Sanz C, Fernández-Garyzábal JF, Cid D. Capsular type diversity of *Mannheimia haemolytica* determined by multiplex real-time PCR and indirect hemagglutination in clinical isolates from cattle, sheep, and goats in Spain. *Vet Microbiol*. 2021;258**:** 109121.
3. Cheng C-M, Lin W, Van KT, Phan L, Tran NN, Farmer D. Rapid Detection of *Salmonella* in Foods Using Real-Time PCR. *Journal of Food Protection*. 2008;71**:** 2436-2441.
4. Giannitti F, Anderson M, Caspe SG, et al. An outbreak of sodium fluoroacetate (1080) intoxication in selenium- and copper-deficient sheep in California. *Vet Pathol*. 2013;50**:** 1022-1027.
5. Hounsome JD, Baillie S, Noofeli M, et al. Outer membrane protein A of bovine and ovine isolates of *Mannheimia haemolytica* is surface exposed and contains host species-specific epitopes. *Infect Immun*. 2011;79**:** 4332-4341.
6. Macías-Rioseco M, Ochoa J, Asín J, Moeller RB, Uzal FA. Salmonellosis in elephants in managed care: report of 2 cases and literature review. *Journal of Veterinary Diagnostic Investigation*. 2023;35**:** 295-299.
7. Maes RK, Grooms DL, Wise AG, et al. Evaluation of a human group a rotavirus assay for on-site detection of bovine rotavirus. *J Clin Microbiol*. 2003;41**:** 290-294.

8. Nyaoke AC, Navarro MA, Beingesser J, Uzal FA. Infectious necrotic hepatitis caused by *Clostridium novyi* type B in a horse: case report and review of the literature. *J Vet Diagn Invest*. 2018;30**:** 294-299.

9. O'Boyle N, Berry CC, Davies RL. Differentiated ovine tracheal epithelial cells support the colonisation of pathogenic and non-pathogenic strains of *Mannheimia haemolytica*. *Sci Rep*. 2020;10**:** 14971.

10. Sasaki Y, Kojima A, Aoki H, Ogikubo Y, Takikawa N, Tamura Y. Phylogenetic analysis and PCR detection of *Clostridium chauvoei*, *Clostridium haemolyticum*, *Clostridium novyi* types A and B, and *Clostridium septicum* based on the flagellin gene. *Vet Microbiol*. 2002;86**:** 257-267.

11. Uzal FA, Hugenholtz P, Blackall LL, et al. PCR detection of *Clostridium chauvoei* in pure cultures and in formalin-fixed, paraffin-embedded tissues. *Vet Microbiol*. 2003;91**:** 239-248.
